# A comprehensive data network for data-driven study of battery materials

**DOI:** 10.1080/14686996.2024.2403328

**Published:** 2024-09-11

**Authors:** Yibin Xu, Yen-Ju Wu, Huiping Li, Lei Fang, Shigenobu Hayashi, Ayako Oishi, Natsuko Shimizu, Riccarda Caputo, Pierre Villars

**Affiliations:** a Center for Basic Research on Materials, National Institute for Materials Science, Tsukuba, Japan; b Research Center for Energy and Environmental Materials, National Institute for Materials Science, Tsukuba, Japan; c Material Phases Data System, Vitznau, Switzerland

**Keywords:** Material databases, battery material, crystal structure, ionic conductivity, capacity, nature language processing, data curation, cathode, solid electrolyte

## Abstract

Data-driven material research for property prediction and material design using machine learning methods requires a large quantity, wide variety, and high-quality materials data. For battery materials, which are commonly polycrystalline, ceramics, and composites, multiscale data on substances, materials, and batteries are required. In this work, we develop a data network composed of three interlinked databases, from which we can obtain comprehensive data on substances such as crystal structures and electronic structures, data on materials such as chemical composition, structure, and properties, and data on batteries such as battery composition, operation conditions, and capacity. The data are extracted from research papers on solid electrolytes and cathode materials, selected by screening more than 330 thousand papers using natural language processing tools. Data extraction and curation are carried out by editors specialized in material science and trained in data standardization.

## IMPACT STATEMENT

We develop a comprehensive data network to accelerate battery material research, integrating multiscale data from three databases and 330,000+ papers using natural language processing and expert curation.

## GRAPHICAL ABSTRACT

**Figure:**
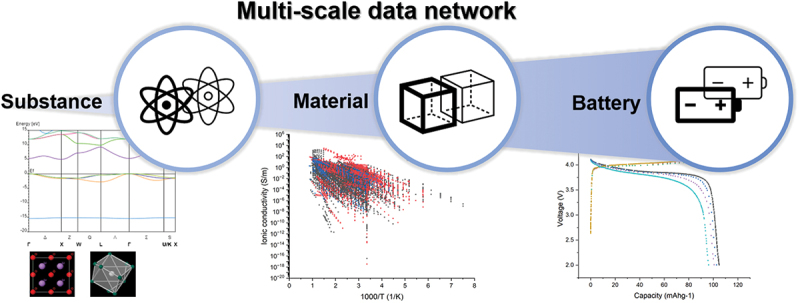

## 1. Introduction

Batteries are a key part of the energy transition and are essential for accelerating the replacement of fossil fuels with renewable energy. To develop batteries with larger capacities, higher performance, and greater safety, innovation in the materials used for electrodes and electrolytes is required. Data-driven materials research is expected to be a new approach that can accelerate the development of new battery materials through materials design and process optimization. Several studies utilizing these methods are referenced in [1–11].

Data-driven studies cannot be conducted without data. What type of data is required for exploring battery materials? The properties of battery materials, such as ionic conductivity and activation energy, depend on their chemical composition, phase composition, and nano- and microstructures. Similarly, their performance in batteries, including charge and discharge capacities, is influenced by the battery’s composition and operating conditions, such as voltage and current. Therefore, to predict the properties and performance of battery materials, data on atomic properties, crystal structures, material structures, and battery performance under various operating conditions are essential. Let’s consider the availability of these data. Atomic properties are readily accessible. Crystal structure data can be sourced from major databases such as ICSD [12] and AtomWork Adv. (AWA) [13]. Additionally, electronic structures, phonon structures, and physical properties can be computed through first-principle calculations using crystal structure data. Some widely-used databases created in this manner include Materials Project [14], AFLOW [15], and NOMAD [16]. However, materials used in batteries are rarely single crystals; they are typically polycrystals, ceramics, or composites. There are few databases focusing on these complex materials, which presents a significant challenge for data-driven studies on practical battery materials. Due to the complexity of these materials, it is difficult to obtain reliable property data computationally, making experimentation the primary method for data generation. Recently, significant advancements have been made in high-throughput experimental techniques aimed at accelerating materials data generation. However, many technical challenges remain, and the variety of materials that can be synthesized and the properties that can be measured are limited. Literature, such as research papers, remains a crucial source of experimental data, as it has been the most common method for materials researchers and engineers to record and present their findings for nearly 200 years. Recently, natural language processing (NLP) techniques have been applied to extract materials data from literature. For instance, Huang [17] has published a database of battery material properties collected using NLP. However, this database only includes composition and properties such as conductivity and battery capacity, but does not contain information on phase composition and structure. Therefore, it is insufficient for materials design purposes.

The aim of this work is to develop a data network from which users can obtain comprehensive data necessary for data-driven studies of inorganic battery materials. This includes data on chemical composition, crystal structures, material structures and properties, and battery performance.

## 2. Data network composition

The battery data network consists of three databases, each dedicated to a specific area and interlinked through an identification system for batteries, materials, and substances:

1. AtomWork-Battery (AWB) [18]: A newly developed database in this work, which includes data on the composition, structure, and properties of battery materials, as well as the performance of batteries made from these materials.
2. AtomWork Adv. (AWA) [13]: A substance database developed by NIMS, containing data on the crystal structure, phase diagram, and properties of single-phase materials.
3. CompES-X [19]: Another database developed by NIMS, which contains data on the electronic structure calculated from the crystal structure data of AWA.

## 3. Substance database AtomWork adv

AWA is a database that contains data on the crystal structure, phase diagrams, and properties of single-phase inorganic materials, meticulously extracted by experts from scientific literature published since 1900 [20]. The phase diagrams feature binary and ternary systems. The property data cover 500 types of properties. Crystal structure data include structure type, space group, Pearson symbol, lattice parameters, atomic coordinates, interatomic distances, and X-ray diffraction patterns. AWA is updated annually. The current numbers of entries are as follows: 47350 phase diagrams, 379,736 crystal structures, and 504,325 property values for 125,606 materials, extracted from 172,392 papers.

**Figure 1.**
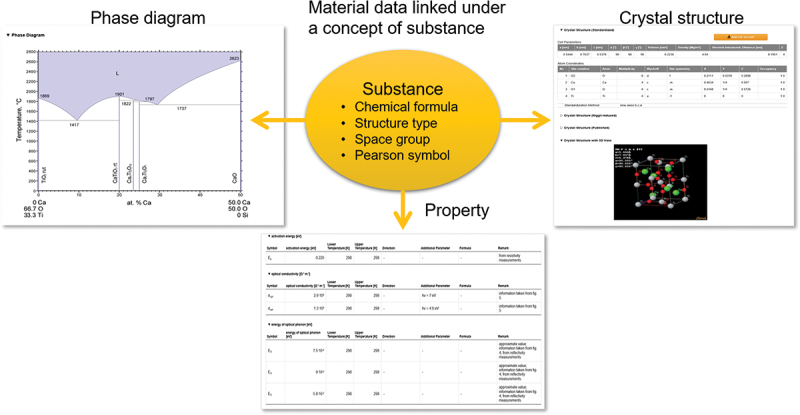
Data structure of AtomWork-adv: interlinking phase diagram, property, and crystal structure data through the concept of substance.

AWA is unique among databases due to a specific feature: despite the fact that the data come from different papers, in AWA, they are interlinked (see Figure 1). Typically, data published in different papers are obtained on different materials. However, some materials share similar chemical compositions and crystal structures, suggesting they likely have similar properties and performance. AWA employs a concept called ‘substance’ to group such materials. A substance corresponds to a distinct phase in a diagram and is defined by five descriptors: chemical system, chemical formula, structure type, space group, and Pearson symbol. The chemical system and chemical formula delineate the substance’s chemical composition, while the other three descriptors define its crystal structure. To illustrate the difference between a material and a substance, consider the following example: a group of materials is reported [21] as Li_1/2−*x*_Sr_2*x*_La_1/2−*x*_TiO_3_ (0<*x* < 0.5). They share the same chemical system (Li-Sr-La-Ti-O), structure type (CaTiO3), space group (Pm-3 m, 221), and Pearson symbol (cP5), but differ in lattice parameter ‘*a*’ based on the value of ‘*x*’. At the material level, they are considered five distinct materials. However, using the substance descriptors, their crystal structures are identical, and AWA uses one representative chemical formula – Li_0.25_Sr_0.25_La_0.42_TiO_3_—as the phase formula to describe their chemical composition, making them identical at the substance level. Similarly, the materials reported in another paper as Li_0.36_La_0.53_Sr_0.03_TiO_3_ are also identified as belonging to the same substance. In AWA, every material is identified by a material ID and every substance by a substance ID, called AWA Material ID and AWA Substance ID, respectively. The concept of ‘substance’ allows AWA not only to interlink data from different papers, but also to provide an interface for external databases to connect to the data within AWA.

## 4. Electronic structure database CompES-X

CompES-X contains data on electronic structures generated by first-principle calculations using the crystal structures in AWA and the calculation software VASP [22]. We developed a framework named Template Oriented Atomic Simulation Toolkit (TOAST) to automatically execute the processes of input data generation, execution of calculations, data analysis, and data conversion. The main data in CompES-X include band structure, density of states, and charge density. Calculations are performed on both non-relaxed and relaxed structures. The current CompES-X contains 28,079 data entries of non-relaxed structures and 27,607 of relaxed structures. AWA and CompES-X are linked to each other through AWA Material IDs, as each crystal structure corresponds to one material.

## 5. Battery materials database AtomWork-battery

AtomWork-Battery (AWB) is a database that compiles data on the synthesis, structure, properties, and performance of battery materials sourced from relevant research papers. Our current focus is on two key categories of materials: solid electrolytes and cathode active materials. We utilize natural language processing (NLP) to identify and retrieve papers pertaining to these material types from a vast array of publications. This technology enables us to extract chemical compositions and property values, and to generate comprehensive overviews. Following this initial selection, papers are meticulously chosen for data extraction, which is carried out by our team of well-trained editors.

### 5.1. Paper screening by nature language processing

**Figure 2.**
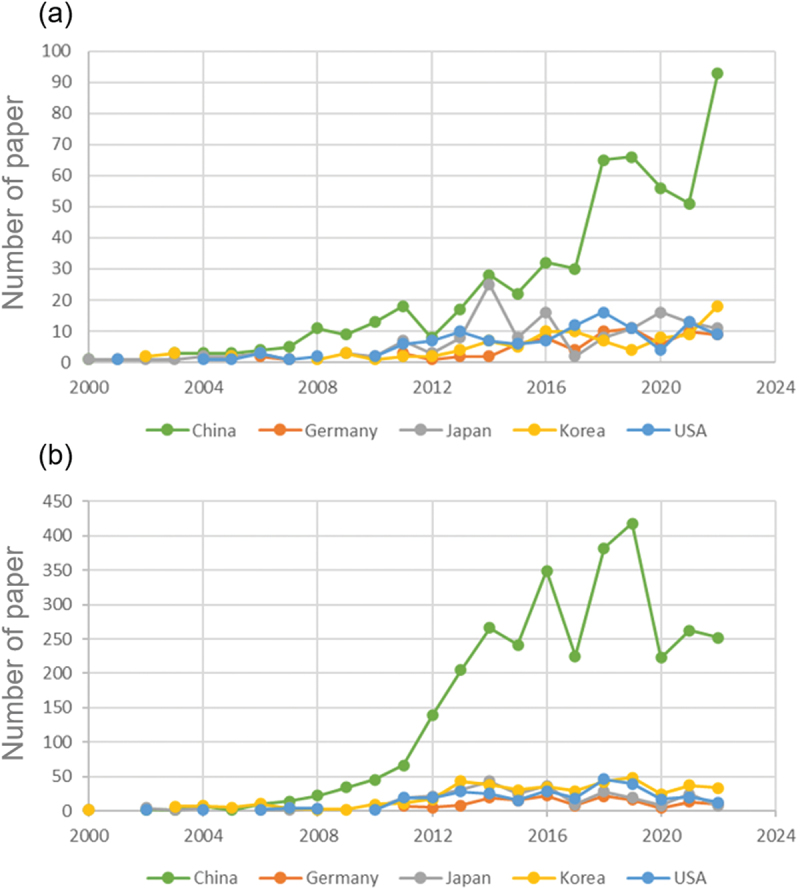
Annual publication output on (a) solid electrolyte and (b) cathode material from the top five countries.

Every year, it is estimated that tens of thousands of papers on battery materials are published. However, a human editor can process only several hundred of them. Therefore, selecting the most relevant and significant papers for data extraction is crucial for compiling a comprehensive dataset efficiently. To identify relevant papers on solid electrolyte and cathode materials, we initially collected several hundred papers for each material type from field experts. Using RapidMiner Studio [23], we then developed a dictionary of feature words frequently found in these papers but less common elsewhere, assigning weights to each word based on its importance. For a new paper, we processed the text in a similar way, and calculated a relevance score by comparing its vocabulary to our dictionary. We set two threshold values, *v*1 and *v*2, with *v*1 > *v*2. Papers scoring above *v*1 are considered relevant, while those scoring below *v*2 are deemed not relevant. Papers scoring between these thresholds undergo further analysis, starting with their titles and abstracts. We defined two sets of keywords – positive and negative. A paper must contain one or more positive keywords and no negative keywords in its title or abstract to be deemed relevant. For solid electrolyte papers, positive keywords include ‘solid electrolyte’, ‘ionic conductivity’, and ‘activation energy’; negative keywords include ‘liquid’, ‘polymer’, and ‘proton conductor’. For cathode materials, positive keywords include ‘cathode’ and ‘positive electrode’, while negative keywords include ‘anode’ and ‘negative electrode’. The source papers [24] used for our NLP tasks are obtained through subscriptions to eight publishers: ACS, AIP, APS, Elsevier, IOP, RCS, Springer, and Wiley. From these publishers we obtain 317 million papers from 354 journals, available in formats such as XML, PDF, and images. Since most of these journals do not focus on battery materials, we narrowed our screening to 19 selected journals, which have published 335,118 papers during 2000–2022. By fine-tuning the threshold values and refining the keyword lists, we achieved a search precision of 80% for solid electrolyte papers and 75% for cathode material papers. The number of papers identified on solid electrolyte and cathode materials is displayed in Table 1. Figure 2 illustrates the annual publication output from the top five countries.

**Table 1.** Overview of papers analyzed, chemical systems, and chemical formulas identified for solid electrolyte and cathode materials using natural language processing tools.

	Number of papers	Number of chemical systems extracted (Li contained)	Number of chemical formulas extracted (Li contained)
Solid electrolyte	1,532	786 (301)	1,335 (608)
Cathode active material	5,095	1,110 (521)	2,422 (1,381)

**Figure 3.**
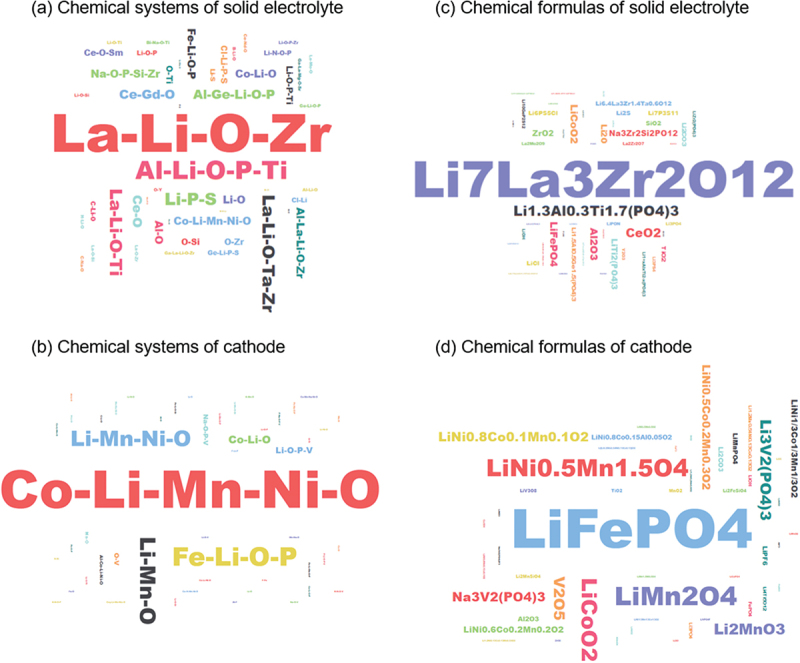
Top 50 most frequently occurring chemical systems and formulas for solid electrolyte and cathode materials, highlighted by font size based on frequency.

We have developed NLP tools to automatically extract chemical compositions and property values from the papers we retrieved. The number of chemical systems and formulas for solid electrolyte and cathode materials is listed in Table 1, and their frequency of appearance is visualized in Figure 3. It is evident that many papers focus on a limited range of chemical compositions, which can lead to data concentration on several well-studied materials if papers are not selected judiciously. Conversely, by carefully selecting papers, we can cover a wide variety of materials while significantly reducing the number of papers needed. By comparing the chemical compositions extracted by the NLP tools with those in the database, we can easily identify which materials are new, and sort the papers by the novelty of the material. Based on these analysis results, we manually select approximately 200 significant papers from each category every year for data extraction according to the following criteria:

1. New chemical compositions are preferred. If the chemical compositions are the same, new chemical formulas are preferred.
2. Property data on ionic conductivity, diffusivity, or capacity must be available, and important experimental parameters should be provided. For example, for capacity data, measurement parameters such as temperature, voltage, current, and cycle number are necessary.
3. Papers containing inconsistent information, unfixable errors, or those deemed unreliable by the editors for any reason, are eliminated during data collection.

### 5.2. Data extraction and data curation

The major challenges in extracting data from literature stem from the diverse content and the various methods used for data processing and representation. Following extraction, data must undergo a process known as data curation, which includes conversion, processing, correction, and generation of data. This stage demands a high level of expertise in material science and data processing skills. In this work, data curation is meticulously carried out by well-trained editors.

#### 5.2.1. Data structure

**Figure 4.**
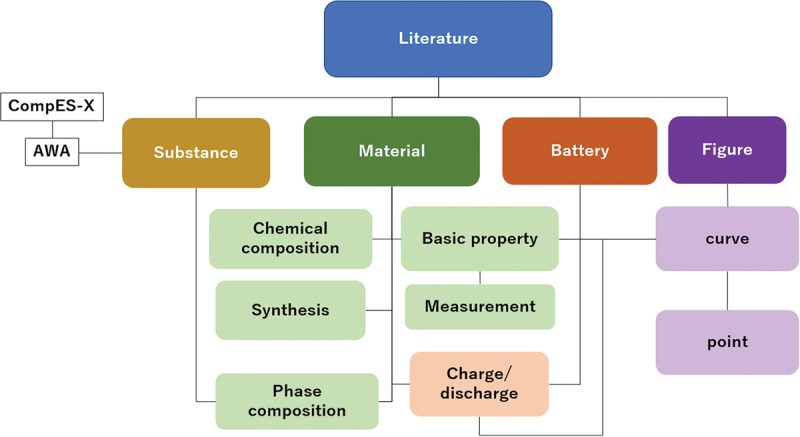
Database structure of AtomWork-battery.

Various data are published in research papers. Aligned with our data collection objectives to design and develop new materials for solid electrolytes and cathodes, we organize this data into a structured format within the AWB database, as depicted in Figure 4. The most critical objects in the database are ‘Material’ and ‘Battery’. The ‘Material’ object encompasses chemical composition, phase composition, synthesis process, and properties, while the ‘Battery’ object includes composition details and charge/discharge capacities. Editors assign the ‘Substance’ object based on the phase composition of the materials, and this object is linked to corresponding substances in AWA. The ‘Figure’ object contains digitized curves of material properties and battery capacities.

#### 5.2.2. Link between substance, material, and battery

Material data in AWB include chemical composition, phase composition, structure, properties, and experimental conditions. Each material is assigned a unique Material ID. For each phase of a material, the chemical composition and crystal structure are determined either from the text description or by analyzing the X-ray diffraction (XRD) data published in the paper. Editors then identify the substance to which the phase belongs, using a process similar to that in AWA. If the substance is also registered in AWA, its AWA substance ID is provided. The linkage to AWA allows us to obtain more detailed information of the substance than what is published in the original papers.

Battery data include compositions of the cathode, anode, and electrolyte, along with charge/discharge capacity and operational conditions. The Material ID of a target solid electrolyte or cathode material is recorded under the material of the corresponding battery component. This organization facilitates the easy identification of specific materials used in a battery and the tracking of batteries that incorporate a particular material.

#### 5.2.3. Property data processing and correction

Property data in AWB includes ionic conductivity, activation energy, diffusion coefficients, and charge/discharge capacity. This data is crucial for materials design, where high precision is essential. However, variations in data processing methods can lead to deviations in property values. For instance, ionic conductivity (*σ*) and activation energy (*E*_*a*_) are two pivotal properties for battery materials. Ionic conductivity is a function of temperature (*T*), typically described by the Arrhenius equation. Most papers present plots of *σ* against T and calculate *E*_*a*_ by fitting these data to an Arrhenius expression. However, there are two expressions are commonly used for fitting:

(1) $$\sigma =\frac{A}{T}\cdot e\left(-\frac{E_{a}^{\prime }}{k_{B}}\cdot \frac{1}{T}\right)$$

(2) $$\sigma =B\cdot e\left(-\frac{E_{a}^{\;\prime \prime }}{k_{B}}\cdot \frac{1}{T}\right)$$

Where *A* and *B* are constants, and *k*_*B*_ represents the Boltzmann constant. The activation energies *E*_*a*_' and *E*_*a*_'', calculated by Equations (1) and (2) respectively, differ and this difference is temperature-dependent. To standardize the data, we digitize all plots from the papers and refit *σ* and *T* using Equation (1) to obtain *E*_*a*_. Additionally, to facilitate comparisons of ionic conductivity at consistent temperatures, we calculate *σ* values at 10-degree increments for all materials. Both the original experimental values and the calculated *σ* values are stored in separate tables in the database.

For capacity data, we extract charge/discharge capacity and related parameters from the text, tables, and graphs in the papers. Each capacity record in AWB includes the value of capacity, operational conditions such as voltage, current, and cycle number, and a link to the corresponding battery. The standard unit of capacity in AWB is mAhg^−1^. When the unit presented in a paper is mAh or mAh cm^−2^, and the mass or mass loading is specified, we convert it to the standard unit. Additionally, if the voltage-capacity curve is available, we also calculate the charge/discharge energy and energy efficiency.

During data digitalization and processing, we find that approximately 10% of papers contain errors in their data. Common mistakes include discrepancies between the data in plots and text, errors in units, and sample-related mistakes. In our data processing, we consistently check the coherence of data across texts, tables, and graphs. If inconsistencies are found, we investigate the reasons and make the necessary corrections.

## 6. Data available for battery materials

**Figure 5.**
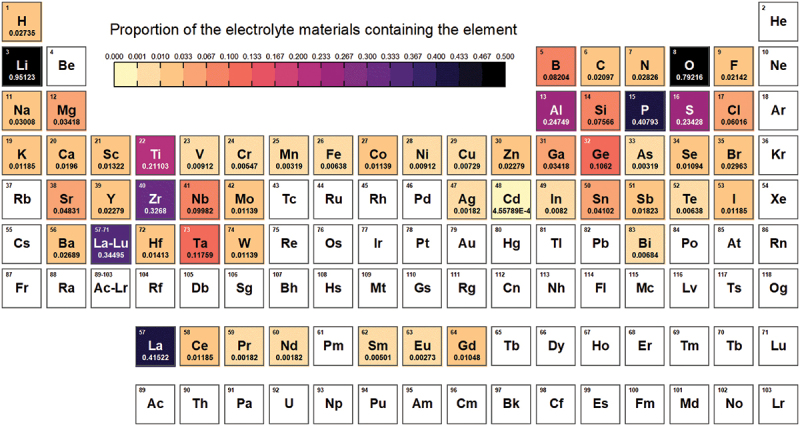
Occurrence of elements in solid electrolyte materials.

**Figure 6.**
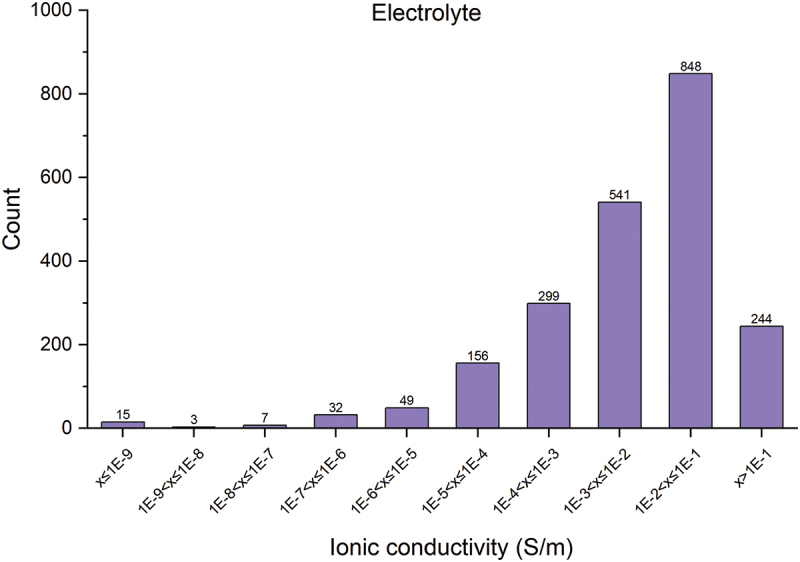
Distribution of ionic conductivity at room temperature in AtomWork-Battery.

**Figure 7.**
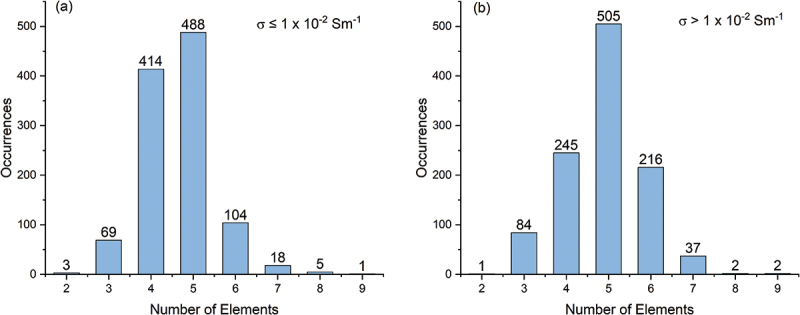
Distribution of number of elements for (a) low ionic conductivity class and (b) high ionic conductivity class materials.

**Figure 8.**
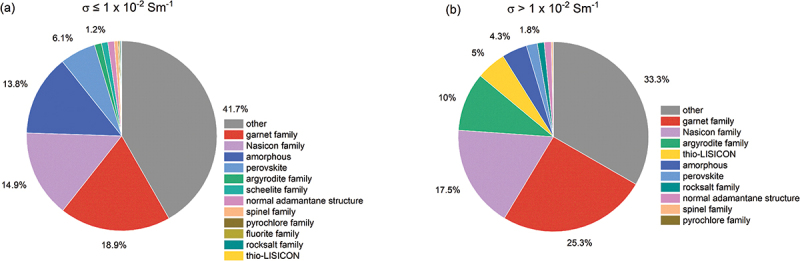
Crystal structure types of (a) low ionic conductivity class and (b) high ionic conductivity class materials.

The current version of the AWB lists data entries as shown in Table 2. Of the 2,712 solid electrolyte materials recorded, there are 461 different chemical systems, with the number of elements ranging from 2 to 9. The elements present in these materials, along with the proportion of materials containing each element, are illustrated in Figure 5. The distribution of ionic conductivities at room temperature is displayed in Figure 6. We categorize the materials into two classes based on their ionic conductivity: a high ionic conductivity class (>10^−2^ Sm^−1^) and a low ionic conductivity class (≤10^−2^ Sm^−1^). The distribution of materials across these two conductivity classes relative to the number of elements is depicted in Figure 7. Materials with more than five elements tend to have a higher likelihood of exhibiting high ionic conductivity. By integrating data from AWA, we can access detailed crystal structure data of these materials. For instance, the crystal structure types of these materials are presented in Figure 8. Although crystal structures like garnet, Nasicon, and perovskite are often highlighted as potential high ionic conductive materials, our statistics do not demonstrate a significant advantage for them; conversely, many amorphous materials also exhibit high ionic conductivity. This suggests that crystal structure type may not be a definitive factor in the search for new ionic conductive materials.

**Table 2.** Number of data entries of current version of AtomWork-battery.

	Solid electrolyte	Cathode material	Total
Paper	538	350	888
Material	2,721	1,122	3,843
Chemical system	461	214	670
Substance	583	252	812
Battery cell	222	1,344	1,566
Material property	23,184	495	23,679
Charge/discharge capacity	1,798	15,667	17,465
Curve	6,504	9,694	16,198
Data point of curve	246,705	770,915	1,017,620

**Figure 9.**
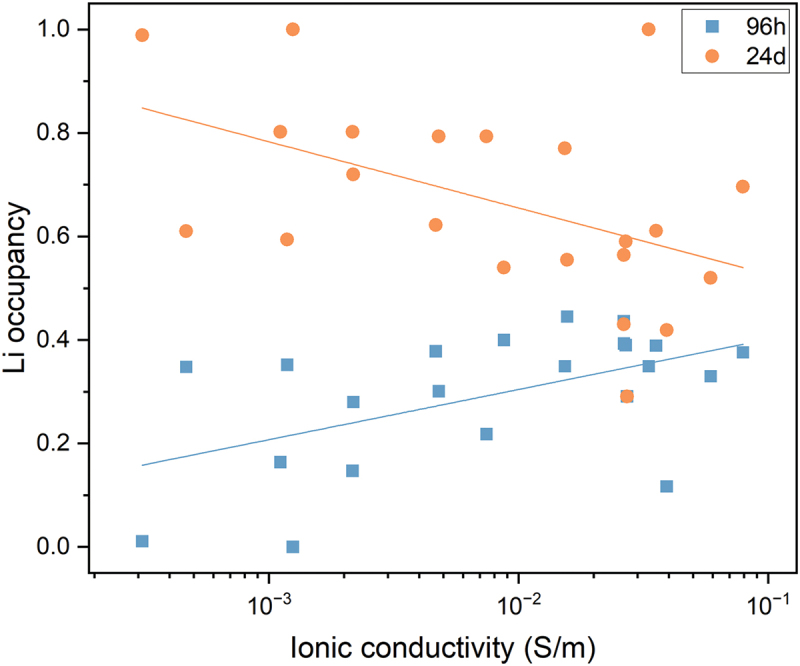
Correlation between Li occupancy at the 96 h and 24d sites and ionic conductivity, showing that higher Li occupancy at the 96 h site is associated with increased ionic conductivity.

Nevertheless, some more detailed structural parameters are found to be strongly relevant to ionic conductivity. For example, ionic conductors with a garnet-type crystal structure have two Li sites, 24d and 96 h. With the ionic conductivity data in AWB and site occupancy data in AWA, we can see that higher Li occupancy at the 96 h site and lower occupancy at the 24d site correlates with enhanced ionic conductivity, as illustrated in Figure 9. This observation is consistent with the computational result [25] on Li_7_La_3_Zr_2_O_12_, which indicate that the 96 h site has lower activation energy, and the experimental result [26] on Li_7−x_H_x_La_3_Zr_2_O_12_, which show that 96 h Li has higher mobility. This example underscores the multi-scale data network’s capability to uncover structure-property relationships that cannot be detected with data from a single database. Additional results will be published in separated papers.

**Figure 10.**
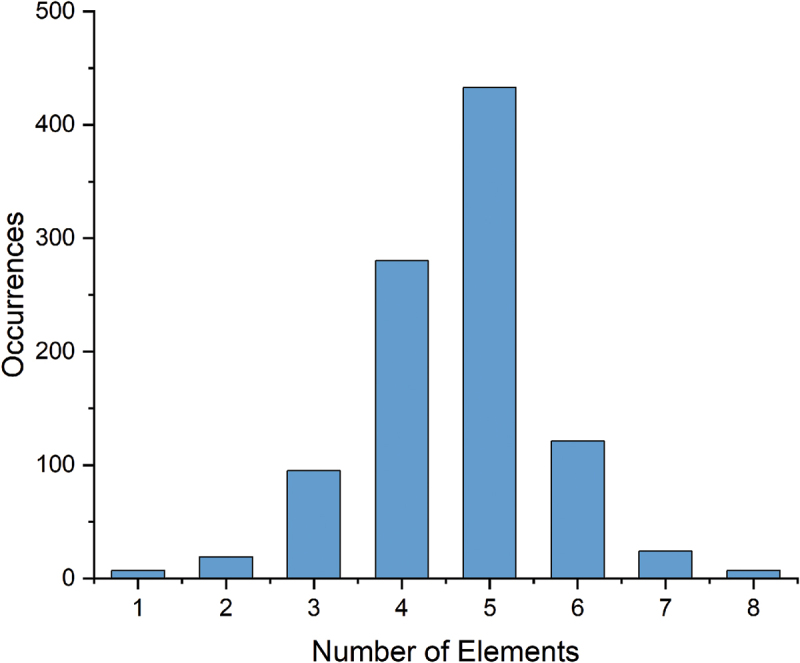
Distribution of number of elements of cathode materials.

**Figure 11.**
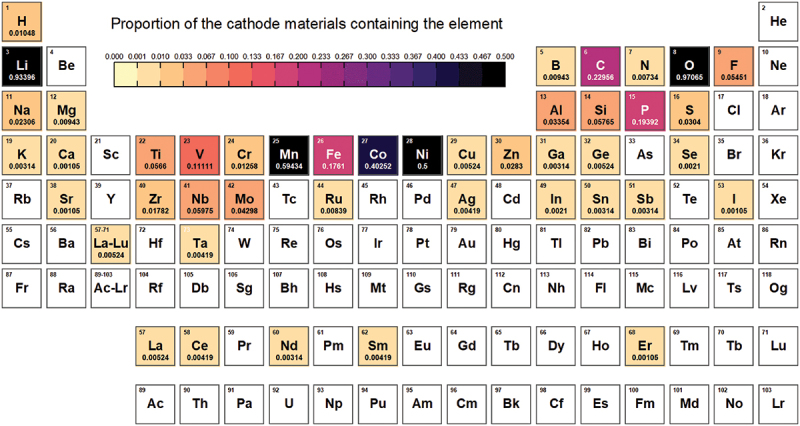
Occurrence of elements in cathode material.

**Figure 12.**
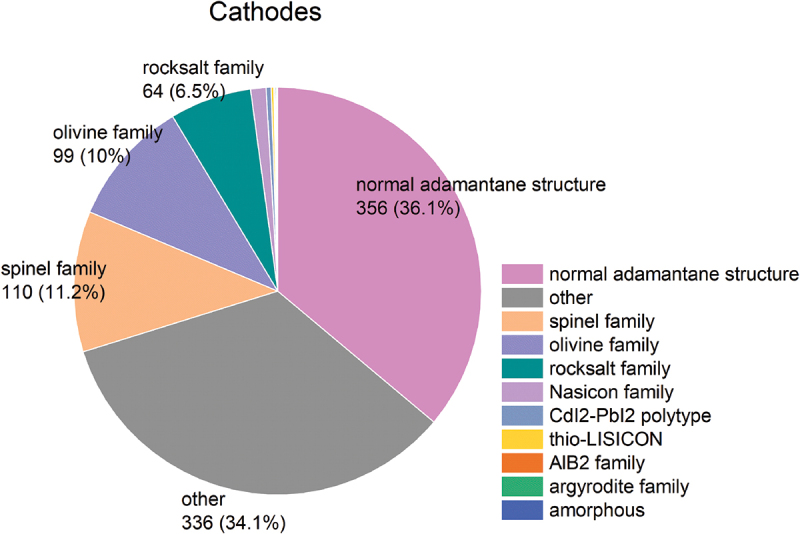
Crystal structure types of cathode material.

**Figure 13.**
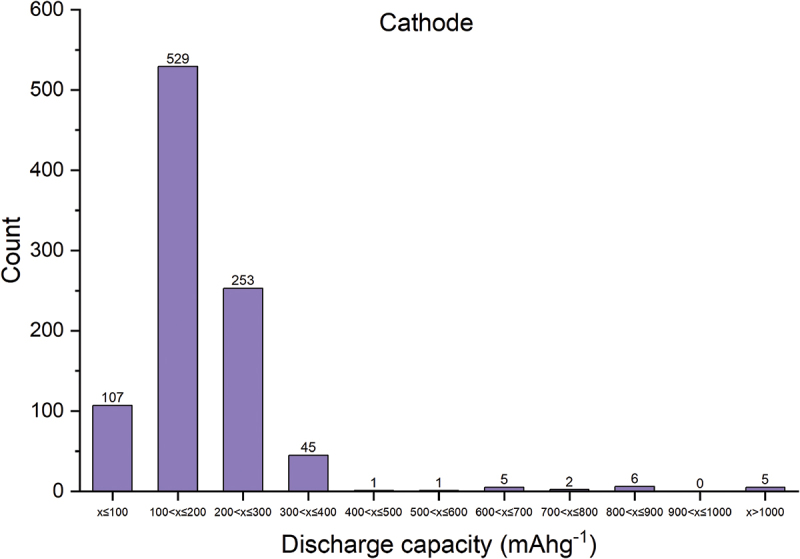
Distribution of discharge capacity of cathode materials in AtomWork-battery.

For the total of 1,122 cathode materials recorded, there are 214 chemical systems with the number of elements ranging from 1 to 8, as illustrated in Figure 10. The quinary system represents the largest proportion. The elements present in cathode materials, along with the proportion of materials containing each element, are illustrated in Figure 11. The structure types and discharge capacities are displayed in Figures 12 and 13, respectively. We do not observe a simple correlation between the number of elements or structure type and discharge capacity. This lack of correlation is considered attributed to the complexity of factors influencing discharge capacity. Prediction of battery capacity likely requires complex models that consider a broader range of factors including material attributes, battery configurations, and operational conditions.

## 7. Conclusion

We have developed an inorganic material data network composed of three interconnected, high-quality databases. These include AtomWork-Battery, which contains data on battery materials; AtomWork-Adv, which focuses on single-phase materials; and CompES-X, which houses data on calculated electronic structures. By integrating these databases, we can compile a comprehensive dataset that encompasses data on substances, materials, and batteries. The data within these databases are continuously updated to include the latest research published in related scientific journals. This data network is designed to be a reliable resource for data-driven studies on inorganic materials for batteries and other applications.
